# Analysis of *Plasmodium falciparum* Rh2b deletion polymorphism across different transmission areas

**DOI:** 10.1038/s41598-020-58300-3

**Published:** 2020-01-30

**Authors:** Yaw Aniweh, Jonathan Suurbaar, Collins M. Morang’a, Prince B. Nyarko, Katherine E. Wright, Kwadwo A. Kusi, Felix Ansah, Eric Kyei-Baafour, Evelyn Quansah, Jessica Asante, Laty G. Thiam, Matthew K. Higgins, Gordon A. Awandare

**Affiliations:** 1 West African Centre for Cell Biology of Infectious Pathogens, University of Ghana, P.O. Box LG54, Legon, Accra Ghana; 2 Department of Biochemistry, Cell and Molecular Biology, College of Basic and Applied Sciences, University of Ghana, P.O. Box LG54, Legon, Accra Ghana; 3 Department of Biochemistry, University of Oxford, South Parks Road, Oxford, OX1 3QU UK; 4 Department of Life Sciences, Imperial College London, London, UK; 5 Immunology Department, Noguchi Memorial Institute for Medical Research, College of Health Sciences, University of Ghana, Legon, Ghana

**Keywords:** Molecular evolution, Conservation genomics

## Abstract

Despite significant progress in controlling malaria, the disease remains a global health burden. The intricate interactions the parasite *Plasmodium falciparum* has with its host allows it to grow and multiply in human erythrocytes. The mechanism by which *P*. *falciparum* merozoites invade human erythrocytes is complex, involving merozoite proteins as well as erythrocyte surface proteins. Members of the *P*. *falciparum* reticulocyte binding-like protein homolog (PfRh) family of proteins play a pivotal role in merozoite invasion and hence are important targets of immune responses. Domains within the PfRh2b protein have been implicated in its ability to stimulate natural protective antibodies in patients. More specifically, a 0.58 kbp deletion, at the C-terminus has been reported in high frequencies in Senegalese and Southeast Asian parasite populations, suggesting a possible role in immune evasion. We analysed 1218 *P*. *falciparum* clinical isolates, and the results show that this deletion is present in Ghanaian parasite populations (48.5% of all isolates), with Kintampo (hyper-endemic, 53.2%), followed by Accra (Hypo-endemic, 50.3%), Cape Coast (meso-endemic, 47.9%) and Sogakope (meso-endemic, 43.15%). Further analysis of parasite genomes stored in the MalariaGEN database revealed that the deletion variant was common across transmission areas globally, with an overall frequency of about 27.1%. Interestingly, some parasite isolates possessed mixed PfRh2b deletion and full-length alleles. We further showed that levels of antibodies to the domain of PfRh2 protein were similar to antibody levels of PfRh5, indicating it is less recognized by the immune system.

## Introduction

Malaria remains a major global challenge with about 216 million cases and 445 000 deaths in 2017^1^. The successes gained by malaria control strategies are threatened by the development and spread of antimalarial drug resistant parasites and insecticide resistant mosquito vectors^2,3^. *Plasmodium falciparum* also exhibits a significant global population structure, with signatures of selection between various populations^4,5^. These high levels of genetic diversity have enhanced the emergence of drug resistance and also hampered the development of effective malaria vaccines^6,7^. The development of a durable and highly effective vaccine remains a key priority in the fight against malaria in an era of renewed global interest in elimination and eradication.

Erythrocyte invasion remains a key target and thus understanding the dynamics of naturally acquired immunity to antigens at this stage will provide a platform for the evaluation of vaccine candidates towards clinical development^8^. Erythrocyte invasion is a multistep process that is rapid and tightly regulated, involving several ligands and their partner receptors such as; erythrocyte binding antigens (EBA 175 & EBA 140) that bind to glycophorin (Gly) GlyA and GlyC, respectively, and erythrocyte binding ligand (EBL-1) that binds to GlyB. The members of the *P*. *falciparum* reticulocyte-binding like homolog family (PfRh1, PfRh2a, PfRh2b, PfRh4 and PfRh5) have been shown to bind to different receptors on the erythrocyte surface^9^, with PfRh4 and PfRh5 binding to complement receptor 1 and basigin, respectively, on the erythrocyte surface^10–14^. The interaction of PfRh5 with basigin on the erythrocyte surface, together with its tripartite interaction with *P*. *falciparum* Cysteine-Rich Protective Antigen (PfCyRPA) and PfRH5-interacting protein (PfRipr)^15,16^, as well as P113 at the N-terminus domain^17^ has emerged as a promising vaccine target^11,12^. However, there remain limitations with PfRh5, such as being less immunogenic^18^ and their absence in non-Laverania species^19,20^, thus necessitating the functional dissection of other key invasion ligands for the purpose of a potential multi-component vaccine.

Previous studies have shown that PfRh2b is processed during schizont maturation and merozoite invasion of erythrocytes^21^. The protein is located in the rhoptry neck but is secreted to the apical end during the invasion process, with its cytoplasmic tail shown to play a role in determining invasion pathways^22^. The PfRh2b gene encodes a 383 kDa protein. A large sequence deletion (~0.58 kb) in the C-terminal region of PfRh2b was reported at high prevalence in *P*. *falciparum* isolates from Senegal^23,24^.

Antibodies against PfRH2b have been shown to block merozoite invasion of erythrocytes^21,25^ and the region containing the deletion is consequently under directional selection enabling those parasites with deletions to evade potent specific antibodies. Growth rate of a 3D7 transgenic strain with the deletion was reported to be elevated relative to wild type parasites^26^, suggesting a possible growth advantage for the mutant parasite *in vitro*. Whilst PfRH2b deletions do not change invasion phenotypes, the combinational vaccine potential of the PfRHs and PfEBAs, necessitate more surveillance to ascertain the proportions of polymorphisms globally to inform downstream decision on any candidate.

The present study, therefore, sought to assess the prevalence of the PfRh2b deletion in clinical isolates from Ghana and across the sub-region using PCR-based genotyping and whole genome sequence data retrieved from the MalariaGEN database, respectively. In Ghana, the copy number of the PfRh2b gene showed multiple copies in areas with high malaria transmission intensity compared with low to moderate transmission areas. We have shown that the deletion frequency in Ghana is estimated to be about 37.3%% whilst globally, it varies significantly between countries. Generally, sub-Saharan Africa has a higher deletion frequency compared with Southeast Asia. Finally, we show that the PfRh5 fold-like domain of PfRh2 is immunogenic, with antibody levels varying with increasing parasitemia.

## Results

### PfRh2b contains a deletion within the C-terminal domain which can be determined by PCR based genotyping

**Figure 1:**
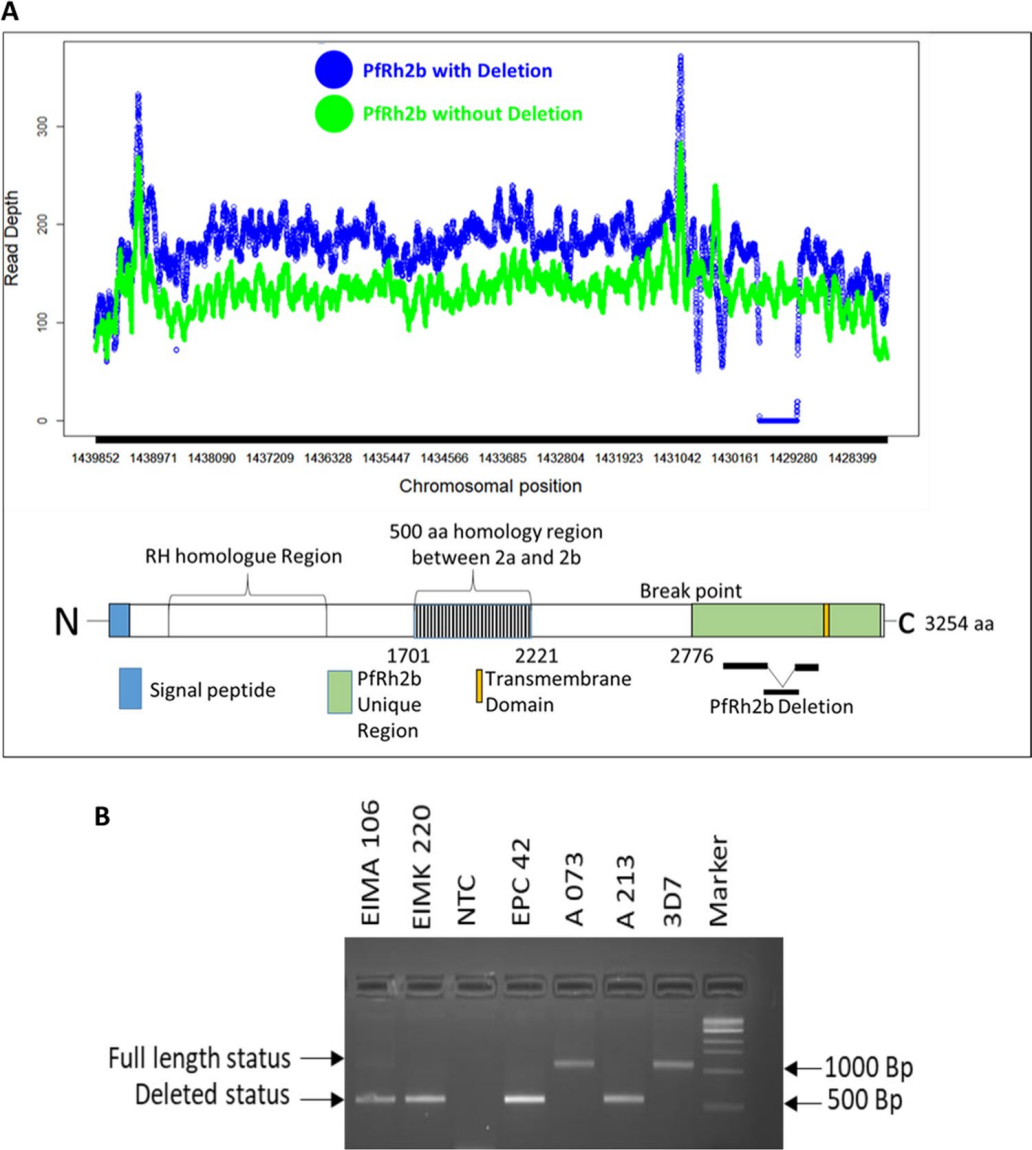
A representation of the PfRH2b deletion mapping. (**A**) The PfRh2b deletion spans approximately (~0.58 kb) on the c-terminal region. Samples without the deletion segment (green) have reads mapping to the segment and genome with the deletion (blue) showed zero reads in the region. The schematic of PfRh2b protein showing the signal peptide (blue), the Rh region, the 2a/2b homology region (shaded), 2b specific region (green), the deletion region and the transmembrane domain (yellow). (**B**) Gel electrophoresis representation of the PfRh2b deletion and full length with the molecular weight indicated (Marker), where EIMA106, EIMK220, EPC42, A073 and A213 are field isolates and 3D7; a laboratory strain of *P*. *falciparum* (NTC – none template control).

With the already described PfRh2b specific deletion described in clinical isolates, we aligned the genomes of the isolates without the deletion and those with the deletion to define the region in our samples. The read depths for the genomes were assessed for the regions (chromosomal position) with a stretch of zero reads (~0.58 kb) by comparing the samples with deletion as observed in the alignment (Supplementary Fig. 2) with samples without the deletion. It was realized that the region corresponding to the deletion fragment mapped exactly on the genomes analysed (Fig. 1A). This was used to define the various regions as well as the extent of the deletion. Since the PfRh2b protein is made up of 3,254 amino acids, having a signal peptide, the RH region, a PfRh2a/2b homology region and a transmembrane domain, the deletion region was mapped unto the schematic (Fig. 1A). PfRh2a and PfRh2b genes vary towards the C-terminus, which is the region shown to harbour the deletion region (indicated as PfRh2b deletion). Both genes have single transmembrane helices which anchor them to the membrane^22^. The deletion regions were found to span from position 9191 to 9770 in the PfRh2b gene with little variation between clones or isolates (Supplementary Fig. 1). To evaluate the frequency of the deletion in PfRh2b, primers flanking the deletion region of the gene were used for the detection polymorphism determination as previously described^24^. The full-length gene gives a PCR product which when resolved on agarose gel (1%) yields a 1000 base pair band, while, the deletion mutant was observed at 500 bps (Fig. 1B) The gel electrophoresis representation of the PfRh2b deletion and full length in Fig. 1B, has EIMA106, EIMK220, EPC42, A073 and A213 as field isolates, 3D7 as a laboratory strain of *P*. *falciparum* and a Non Template Control (NTC).

### Deletion polymorphism of PfRh2b is present at high frequency in parasite populations from Ghana

**Figure 2:**
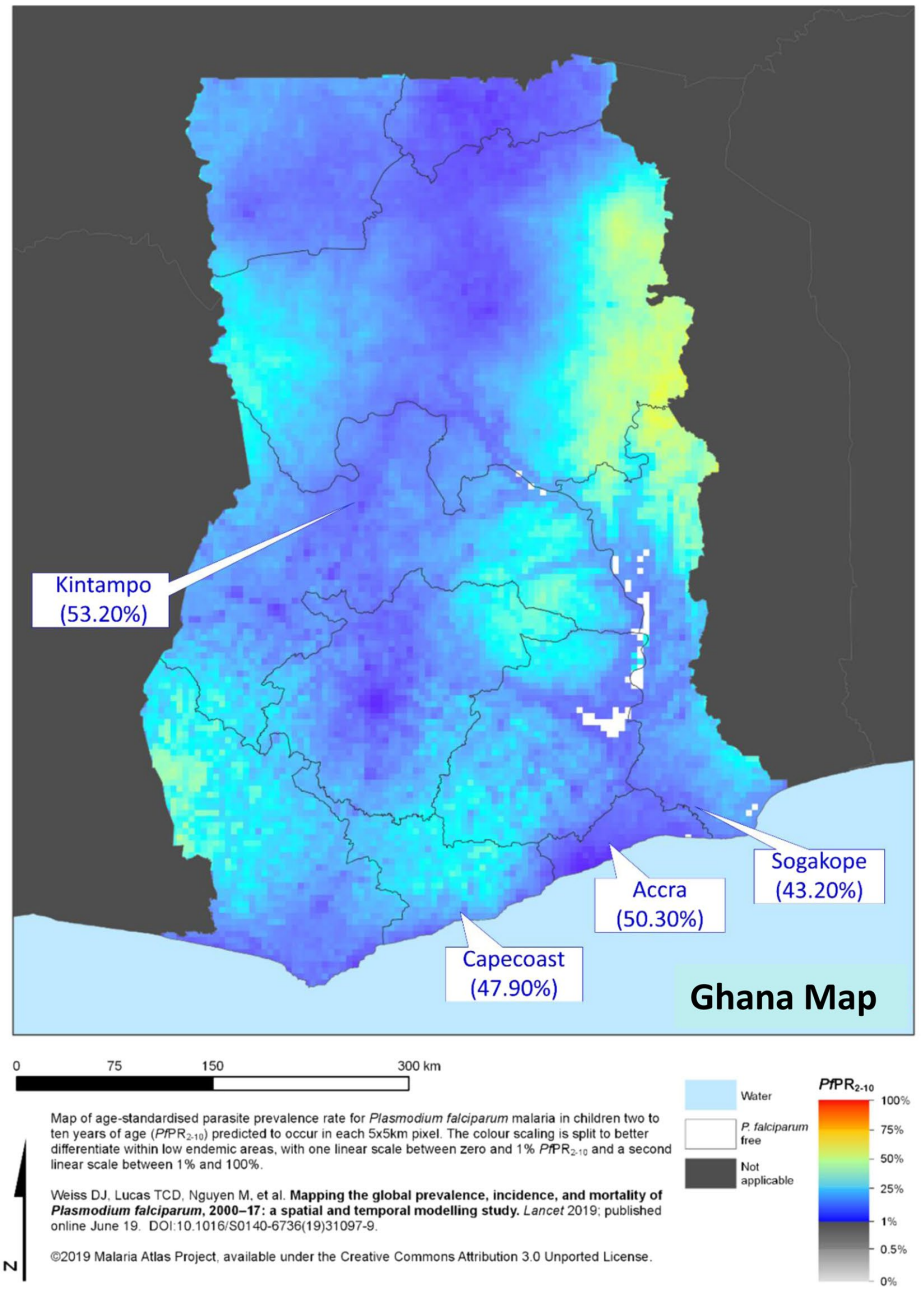
PfRh2b deletion frequencies map of Ghana. A map of Ghana showing the geographical locations of the areas that parasites were sampled from. Frequencies of PfRh2b deletion mutants in parasites from low transmission sites in Ghana (Sogakope (n = 197), Cape Coast (n = 618) and Accra (n = 155)) were compared to those in a high transmission area (Kintampo (n = 248)).

To determine the frequencies of the deletion polymorphism across the different transmission areas in Ghana, 1,218 *P*. *falciparum* clinical parasite isolates obtained from Accra (low transmission), Cape Coast (moderate transmission), Sogakope (moderate transmission) and Kintampo (high transmission) were genotyped. Generally, a high proportion of PfRh2b deletion (37.3%) was observed in all the Ghana data analysed. This trend was seen in all the areas where samples were collected irrespective of the endemicity level (Supplementary Table 1). Comparing the frequency of the deletion across sites, parasites from Kintampo showed the highest frequency (53.20%), followed by Accra (50.30%), Cape Coast (47.90%) and Sogakope (43.15%) (Fig. 2), though these differences were not statistically significant. A small proportion (73/1218) of samples were found to harbour both the deletion mutant and full-length gene, indicating possible mixed clones. This mixed status was most observed in Cape Coast followed by Kintampo, and then Accra (Supplementary Table 1, P > 0.05). We further confirmed this by clonal diversity analysis of the samples using MSP-1 gene typing. Some of the samples were confirmed to contain multiple clones (Supplementary Fig. 1). The data presented provides evidence that there is an ongoing selection or deletion acquisition in the field. This raises interest in the possible importance of the deleted domain immunologically.

### Frequency PfRh2b deletion varies across different endemic areas

**Figure 3:**
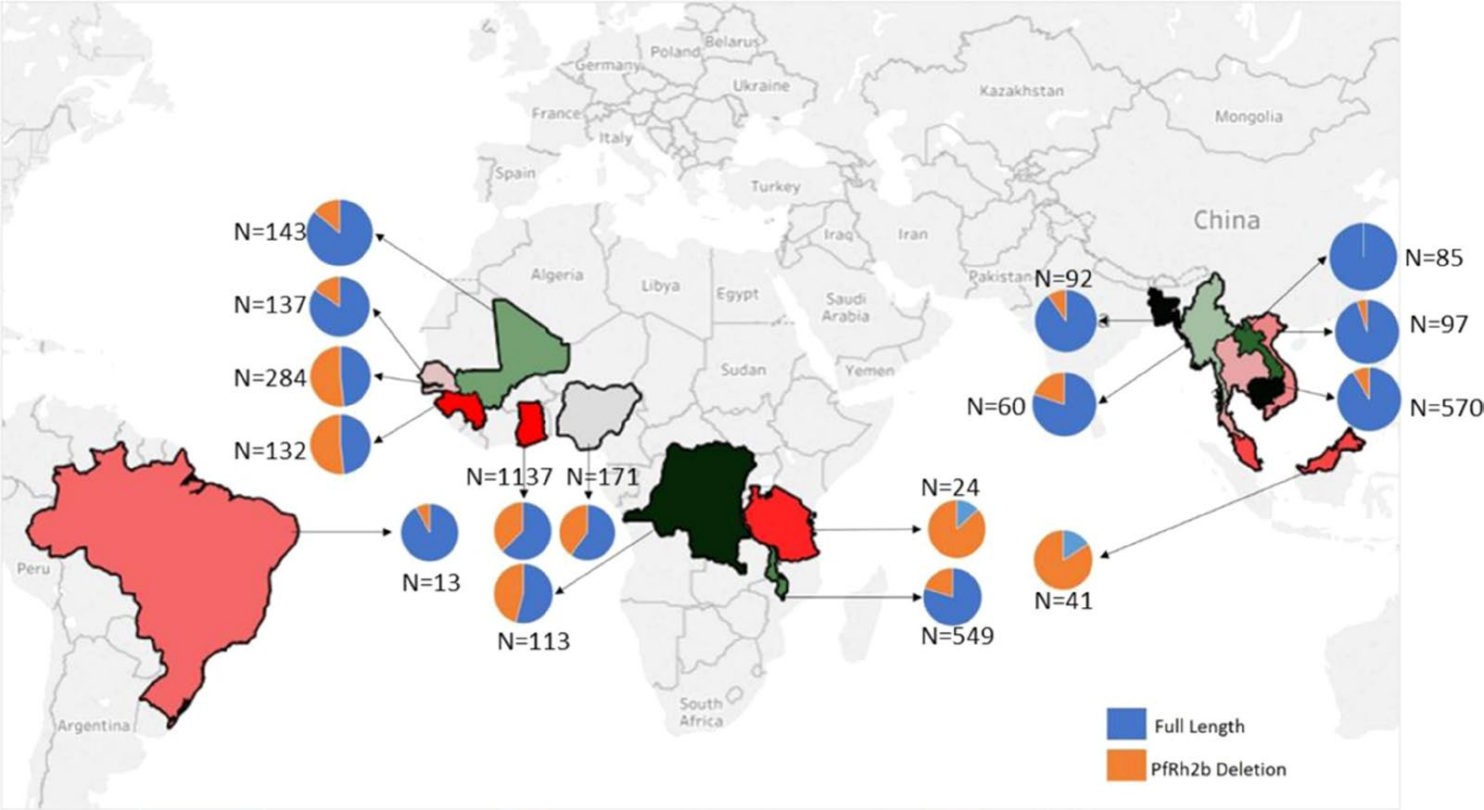
Deletion frequency of PfRh2b in 16 countries in the world. Frequencies of PfRh2b deletion mutants in five west African countries (Guinea/The Gambia, Senegal, Mali, and Ghana), Central and East Africa countries (Tanzania and Malawi), three South East Asia countries (Bangladesh, Laos, Cambodia, Vietnam, Thailand, and Malaysia) and one country in south America (Brazil) were determined using data from MalariaGEN and published articles; n = number of samples.

The high prevalence of the PfRh2b deletion in Ghanaian *P*. *falciparum* clinical isolates prompted the assessment of the current status of this deletion in other malaria endemic countries within and outside Africa. Globally, PfRh2b deletion was found in all selected countries. The frequency of the deletion of PfRh2b was varied, with Tanzania (87%), The Gambia/Guinea (51.8%), Malawi (58%), DR Congo (46%), Nigeria (40%) and Ghana (37.3%) showing a greater percentage of the deletion (Supplementary Table 2) whilst the other African countries; Senegal (15.3%) and Mali (14.0%) harboured lower numbers of PfRh2b deletion. In addition to data generated in the context of this study, published data^24,23^ were used to obtain a global view of the PfRh2 deletion status (Fig. 3), from which it was observed that other than, Malaysia (84.0%), the rest of Southeast Asian countries (Bangladesh (9.8%), Cambodia (8.6%), Thailand (14.2%) and Vietnam (5.2%) harboured deletion of PfRh2b with frequencies less than 20% whilst Laos had no deletions detected. South America (Brazil) showed low PfRh2b deletion frequency of 8.0%.

### Parasite populations possessing full-length PfRh2b showed duplicated gene copy number in high transmission areas

**Figure 4:**
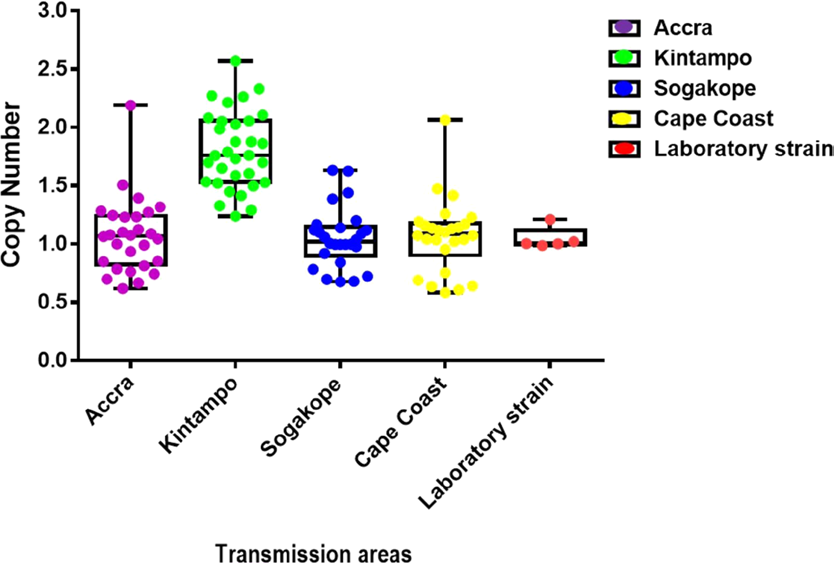
Copy number variations (CNV) of PfRh2b in two different malaria transmission sites (Accra and Kintampo) and clonal diversity of the PfRh2b. PfRh2b gene copy numbers of parasites Accra (n = 72), Kintampo (n = 72), Sogakope (n = 62) and Cape Coast (n = 67). were determined using real time quantitative PCR. Controls laboratory strains (Dd2, 3D7, W2mef, K1 and Nf54) were also tested. Data are presented as box and whisker plots for the different communities, the line through the box is the mean/median.

The high frequency of PfRh2b deletion mutants observed in most parts of the world (Fig. 3) raised the possibility that this deletion could be involved in parasite adaptation and immune evasion. Also, with PfRh2b shown to have high copy number variation (CNV) frequencies in African parasite population^27^, it was of interest to investigate CNV that exist in *P*. *falciparum* parasites isolates from Ghana. Out of 72 isolates with full length gene analyzed, 13 (18%) were found to have double copies of the gene. Of note, parasites obtained from Kintampo had a higher overall gene copy number compared to those from the lower transmission areas (Accra, Sogakope, and Cape Coast) (Fig. 4). Laboratory cultured *P*. *falciparum* strains (Dd2, 3D7, K1, HB3 and W2mef) were found to harbour single copies of the PfRh2b gene.

### Antibodies to PfRh5 and PfRh2b domain antibodies persists through dry and wet seasons

**Figure 5:**
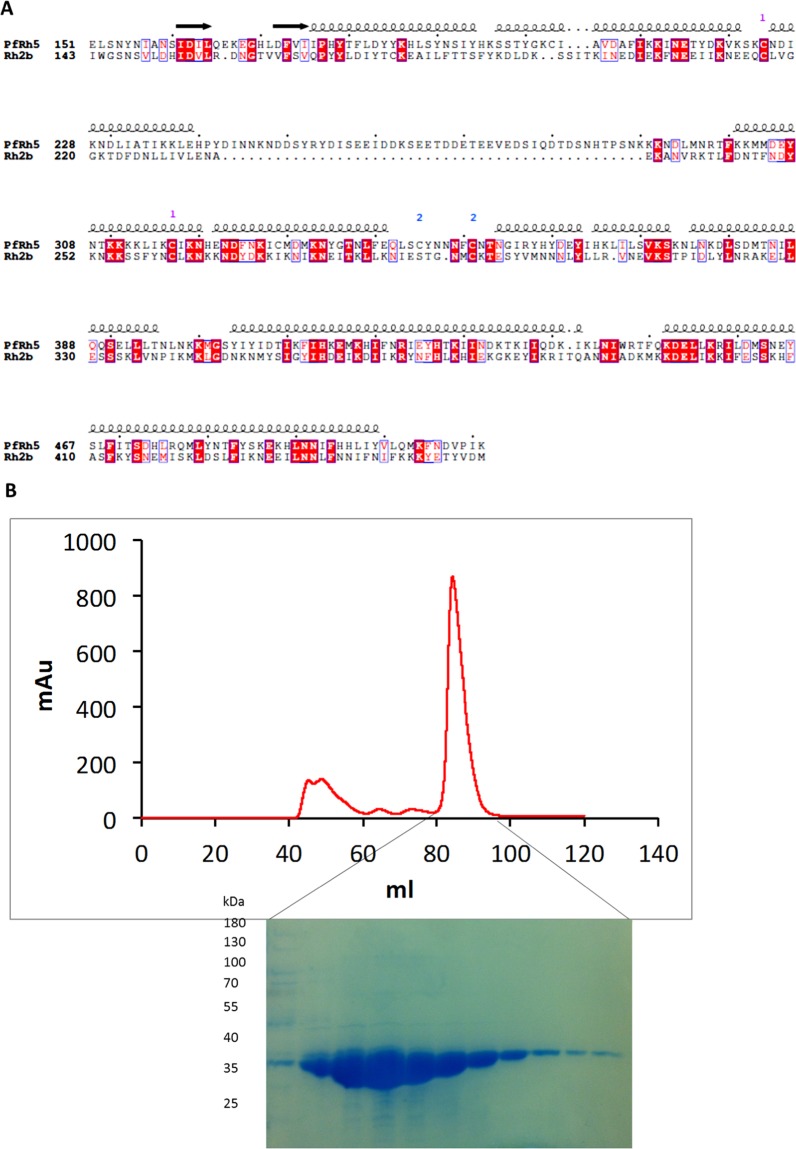
PfRh2 domain sequence alignment and purification. (**A**) Amino acids alignment for PfRh5 (151–512) and PfRh2 (143–450). The alignment showing the conserved amino acids shaded red, whilst similar amino acids indicated in red. The possible secondary structure is indicated over the sequences; arrow (beta sheets), coil (helices). Gaps have been shown by dots (**B**) Purification of PfRh5-like domain of PfRh2. After Ni^2+^-NTA chromatography, the protein was applied to a Superdex 200 16/60 column (UV280 trace shown at top), and the fractions run on an SDS-PAGE gel and stained with Coomassie (bottom). The expected molecular weight is approximately 37 kDa.

**Figure 6:**
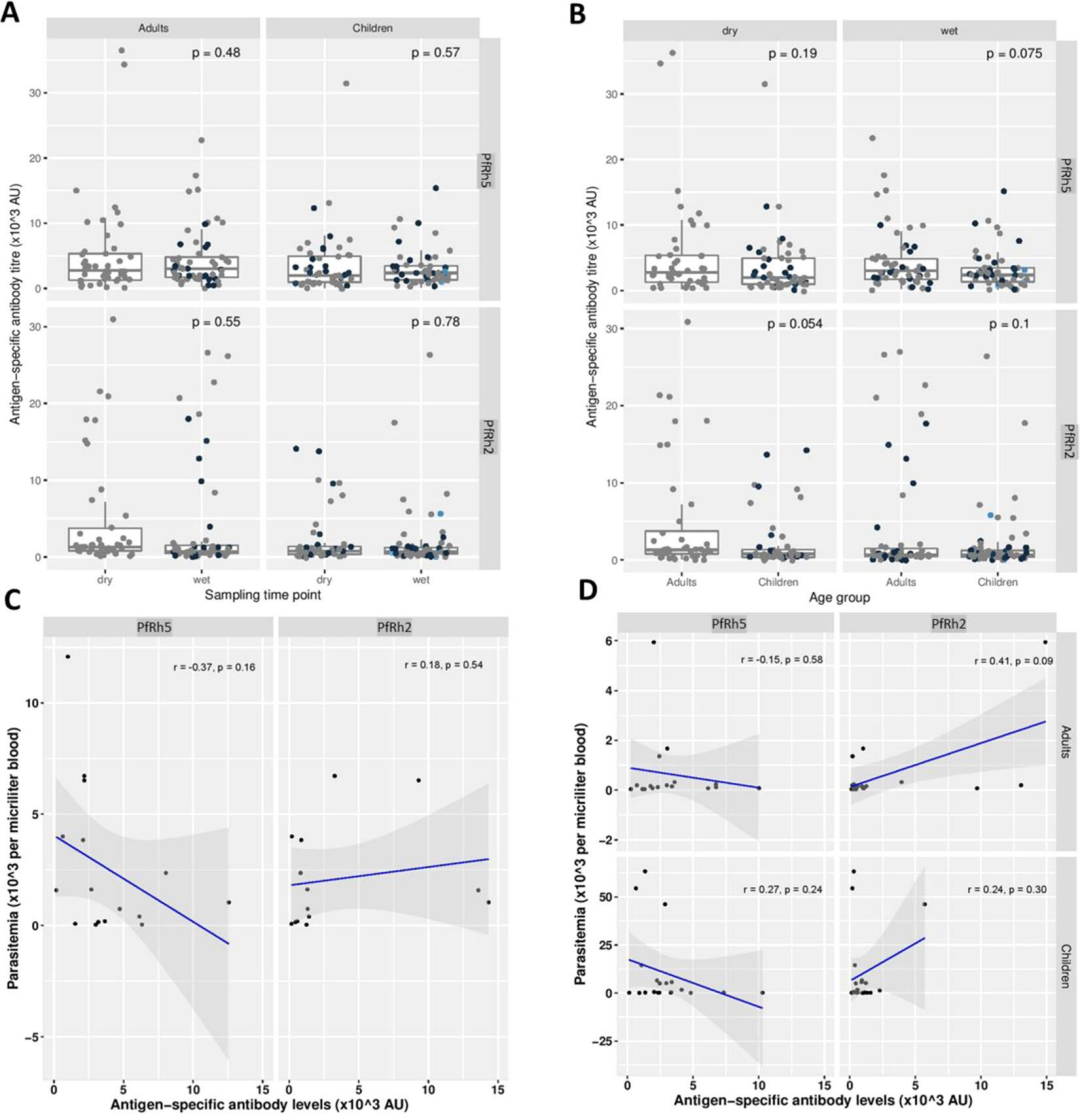
Antigen-specific antibody titres of PfRh5 and PfRh2b (PfRh5-like domain) as well as the effect of seasonality, age and parasitemia on the antibody responses. (**A**,**B**) Antibody titres for PfRh5 and PfRh2b were not significantly different (P > 0.05) in the two age groups (children and adults) in both the dry and wet season. (**C**) The correlation coefficients between parasitemia (×10^3^ per milliliter of blood) against antigen specific antibody levels (×10^3^ AU) for PfRh5 (r = −0.37, p = 0.16) and PfRh2 (r = 0.18, p = 0.54). (**D**) Correlation coefficients between parasitemia (×10^3^ per milliliter of blood) against antigen specific antibody levels (×10^3^ AU) for PfRh5 and PfRh2 in both adults and children.

The possible structural similarity between the PfRh2b domain and the PfRh5 proteins^14^, prompted us to measure the levels of antibody response to the PfRh5-like region of PfRh2b. The domain of PfRh2b; using the amino acid sequences (residues S146-T450) of PfRh2b was aligned with the solved structure of the PfRh5 domain^14,28^. The level of conservation observed was approximately 20% (Fig. 5A). This construct was recombinantly expressed with a C-terminal hexa-histidine tag in Drosophila melanogaster S2 cells and purified to homogeneity (Fig. 5B). An enzyme-linked immunosorbent assay was performed to determine the plasma levels of antibodies to the PfRh2b domain and the PfRh5 protein in malaria-exposed individuals in Ghana with varying ages, who were sampled in both the dry and wet (rainy) seasons. It was observed that there were no significant differences in both PfRh5 and PfRh2b antibody levels in the cohort for both dry and wet seasons in adults (>14 years) and children (1–14 years) (Fig. 6A,B), however, the levels of antibodies to each of the antigens tested persisted from the dry to the wet season. On evaluating the relationship with parasitemia in the cohort studied, it was observed that, generally, there was a non-significant negative correlation between parasitemia and antibody level for the Rh5 protein (r = −0.37, p = 0.16). Antibodies levels to the PfRh2 antigen however showed non-significant positive correlation (r = 0.18, p = 0.54) with parasitemia. On stratifying the data into age groups, children and adults, the trends remained same for PfRh5 and the PfRh2 protein between parasitemia and antibody levels (Fig. 6C,D).

## Discussion

Different polymorphisms have been shown to impact the role and usage of the different ligands of the PfRh family. Copy number polymorphism in the PfRh1 gene was associated with the use of the sialic-acid dependent invasion pathway in laboratory strains of *P*. *falciparum*^29^. However, no changes in PfRh1 copy numbers were observed in field isolates, suggesting that the variation in lab isolates was not naturally occurring but likely induced by *in vitro* culture conditions^29^. Copy number variations are more common in the PfRh4 gene^30^, whereas PfRh5 shows very limited polymorphisms overall, with no evidence of number variation.

The large deletion in the 3’ end of the PfRh2b gene has been thought to be an immune evasion strategy used by the parasite but comes at the cost of decreased invasion efficiency in neuraminidase (Nm) treated erythrocytes^31^, demonstrating borderline impact on invasion efficiency into Nm-treated cells^23^, but a significant increase in invasion into trypsin treated cells^32^. However, these associations were not confirmed when testing the role of the deletion on invasion efficiency in an isogenic background^26^. In this study, we hypothesized that if the deletion polymorphism was indeed an adaptation by the parasite in response to immune pressure then the frequency of the deletion would increase with increasing transmission intensity. Our results showed that although the highest frequency was found in Kintampo, a high transmission setting, it was also high in Accra, where transmission intensity was lowest. This suggests that the mechanisms underlying the occurrence of the deletion are more complex than immune pressure alone. Additional analyses broaden the investigations to the global level by determining the frequencies of the PfRh2b deletion in parasite genomes stored in the MalariaGEN database, including isolates from Ghana, Nigeria, DR Congo, Cambodia and Vietnam^24^. Consistent with the pattern described previously, the frequencies of the deletion were generally higher in the African isolates compared to parasites from South East Asia and South America.

The PfRh2b gene had previously been shown to be more liable to copy number variation with parasite populations from Africa (The Gambia and Malawi) shown to have the highest frequency (>50%) of the gene expansion compared to parasite isolates from South East Asia (Lao, Cambodia and Thailand) and South America (Peru and Venezuela)^27^. With this observation, we investigated the copy numbers of the gene in Ghanaian parasite isolates and evaluated intra-country distribution. It was observed that a doubling in copy numbers of the PfRh2b gene occurred in the high transmission area, relative to the low to moderate transmission communities in Ghana. The mechanism driving this phenomenon is not clearly understood yet. It is also not clear if the gene duplication is related to the high prevalence of the deletion mutant. This calls for increased surveillance for both the deletion mutant and CNV, to better understand if these are related or elicited by entirely independent mechanisms. *In vitro* analysis has indicated the deletion affect growth rate in a laboratory adapted strain^26^. Recent forward genetics study on the PfRh2b gene has shown it to play a role in in alternative pathway selection. However, the impact of CNV on invasion function of the gene is yet to be determined.

The PfRh2 receptor binding region had been narrowed down to the N-terminal domain^33^. Since the PfRh2 proteins are large in molecular weight, any effect on the receptor binding domain could drive a down-stream effect making other domains accessible or not. Structurally, the modelled PfRh5-like domain of PfRh2 had been shown to be very similar to the solved PfRh5 structure, with some conservation in residues^14,28^. By the resolved structure of PfRh5, it has been described to be composed of three helical-bundles coming together to form a kite-like structure, similar to the predicted PfRh2 domain^14^. With this structural similarity, we continued to evaluate the ability of this domain to stimulate antibodies in natural patients relative to PfRh5 protein during the wet and dry seasons, when malaria transmission is high and low, respectively. Generally, it was observed that both the PfRh5-like domain of PfRh2 and PfRh5 showed similar antibody levels. The low antibody levels for the PfRh2 domain could be as a result of it not being well exposed in natural infections as well as being within the region that harbours the receptor binding domain^25,33^. Like PfRh5, the PfRh2 domain showed antibodies that persisted through the two seasons, whilst there was no age dependent variation. The data presented here, thus, reinforce PfRh2b as a protein that immunogenic, which has been shown to be important for protection against infections and reducing parasite multiplication^24,34^.

## Methods

### Study sites and sample collection

The study used samples from a cohort study with ages ranging from 1 to 70 as well as a cross-sectional study. Ethical clearance was obtained from the Noguchi Memorial Institute for Medical Research (NMIMR-IRB CPN 004/11–12), Kintampo Health Research Centre (KHRCIEC/FEA/2011-13) and Ghana health Service (GHS-ERC:12/05/12). All methods were performed in accordance with the relevant guidelines and regulations as proposed in the protocol for the study. Informed consent was obtained from all patients and/or guardians before samples were taken. Three distant locations in Ghana – Accra, Cape Coast, Sogakope and Kintampo- with different transmission intensities were selected for sampling. Venous blood of 1021 *P*. *falciparum*-infected (detected by microscopy and RDT), people were sampled spanning the 2015–2018 malaria transmission seasons. Specifically, 155 parasite isolates from Accra (capital city), which has a relatively low transmission intensity (entomological inoculation rate (EIR) <50)^35^, 248 parasite isolates from Kintampo (420 Km from Accra), considered holoendemic with EIR of >250^36^ and 618 samples from Cape Coast (140 Km from Accra), considered a meso-endemic location (EIR undetermined) were used. All sample processing and storage was uniform to ensure comparability from the three different locations.

### DNA extraction and parasite genotyping

QIAmp DNA Minikit (Qiagen, Valencia, CA, USA) was used to extract DNA from whole blood by following the manufacturer’s protocol, and DNA was stored at −20 °C. PCR-based 18sRNA genotyping was performed on all samples to obtain only *P*. *falciparum* positive samples as earlier described^37^.

### PfRH2b deletion genotyping

For the PfRh2b deletion genotyping, a hemi-nested PCR as previously described^24^ was used with the following modified cycling conditions: initial 3 minutes (mins) denaturation at 94 °C followed by 30 cycles with 30 seconds (s) at 94 °C, 40 s at 55 °C and 1 minute (min) at 68 °C and 7 mins final extension at 68 °C for both outer and nested PCR. The amplification utitlised the following primers in the assay: outer PCR: (forward primer: 5′-TAATGATATAAAGGATCTTGGTGA-3′, reverse primer: 5′-AGGAAATCATCCA TTTTGTTATGGT-3′), for the hemi-nested second PCR, (forward primer: 5′-GGATAAAATACT AGAAGGAAGTGA-3′, reverse primer: 5′-AGGAAATCATCCATTTTGTTATGG T- 3′). Amplicons were separated and visualized on a 1.5% ethidium bromide stained agarose gel using the GE Imager (GE Healthcare). Also, the amplicons were sequenced by Sanger sequencing method using the following primers 5′-GGATAAAATACTAGAAGGAAGTGA-3′ and 5′-AGGAAATCATCCATTTTGTTATGGT-3′. Using Benchling, consensus sequences were generated and viewed by Jalview.

### MalariaGEN data retrieval and analysis

The *P*. *falciparum* whole genomes were retrieved from Pf3K MalariaGEN database (https://www.malariagen.net/apps/pf3k/release_3/index.html). Genomic locations of PfRh2b were obtained from PlasmoDB database (http://plasmodb.org/plasmo/app/record/gene/PF3D7_1335300). First, Samtools^38^ view option was used to extract reads for the PfRh2b region, followed by generation of consensus sequence using a custom made bash script involving mpilup option, bcftools, vcftools^39^, as well as seqtk tool. Sequences with gaps or non-specific mappings were viewed using Artemis^40^ and removed using custom made python scripts. The Sanger sequence amplicons of the PfRh2b gene and the Pf3K genomes consensus sequences were aligned alongside the 3D7 PfRh2b sequence using MAFFT^41^. Aligned sequences were viewed using Jalview^42^, focusing on the expected PfRh2b deletion region. Secondly, Bedtools^43^ was used to extract the read depths for the individual chromosomal position for the PfRh2b gene in the bam files using the genomic region of the gene. The read depths were analyzed using R-programming software (https://www.r-project.org/) to generate the deletion frequencies on samples containing the deletion from West Africa and Southeast Asia. The R-programming software was also used to generate a graphical representation of the deleted segment on the PfRh2b gene.

### Copy number variation (CNV)

CNV was determined using real-time PCR run on a Quant Studio 5 system (Applied Biosystems). PCR cycling conditions were optimized for *P*. *falciparum* DNA. Data acquisition was done at the end of elongation of each cycle. Specificity of amplification was ascertained by melting-curve analysis of each PCR product. The primers used in the reactions are; P2 5′-GCGGATCC CAACAACAAAGAAATATCCAAG-3′, P3 5′-GCGAATTCTTAATCATGTGTACTAGACG TGTTTC-3′, seryl-tRNA synthetase primers were F: 5′-AAGTAGCAGGTCATC GTGGTT-3′ and R: 5′-TTCGGCACATTCTTCCA TAA-3′. All reactions were performed at primer concentrations of 0.5 μM, 1X PerfeCTa SYBR Green Supermix (Quantabio) and 2 μl of DNA template. Each sample was run in triplicate using the QuantStudio5 (Applied Biosystems) real-time PCR machine. The cycling conditions were an initial 50 °C for 2 mins and 95 °C for 1 min followed by 40 cycles of 95 °C for 30 secs, 54 °C for 40 secs and 60 °C for 1 min and a melt curve stage of 95 °C for 15 secs, 60 °C for 1 min and 95 °C for 1 sec. The seryl-tRNA synthetase was used as the endogenous control as well as setting up a non-template negative control. The copy numbers for the samples were determined and repeated for certainty. The melting curve analysis was performed for each run and experiments with non-specific products, Ct > 32 or standard deviation (SD) ≥ 0.5 were repeated. The delta delta Ct formula (2^−ΔΔCt^) was used to estimate the relative copy numbers.

### Assessment of clonality

Polymorphisms in MSP-1 and MSP-2 were used to assess clonality and to ascertain mixed infection as described previously^44^. All PCR reactions included DNA of laboratory strains 3D7, HB3, 7G8, W2mef and Dd2 as positive controls.

### Generation of recombinant antigen

Recombinant PfRh5 was expressed in S2 cells and purified as previously described^14^. The synthetic gene encoding residues S146-T450 of the 3D7 PfRh5-like domain of PfRh2b was codon-optimised for expression in *Drosophila melanogaster*–derived cells and designed to include a Kozak sequence and EcoRI site at the 5′ end and a His tag and a NotI site (Life Technologies). The gene was subcloned into the pExpreS2-1 vector (ExpreS2ion Biotechnologies, Horsholm, Denmark). The PfRh2b construct was transfected into Schneider (S2) cells using ExpreS2 Insect-TRx5 (ExpreS2ion) transfection reagent and selected over a period of three weeks with 1.5 mg/mL zeocin, in EX-CELL 420 insect cell medium supplemented with L-glutamine (Sigma) and 10% fetal calf serum (Gibco). The resulting stable, polyclonal cell line was grown in EX-CELL 420 medium with no fetal calf serum or zeocin, and the cells harvested after 4 days. The cell culture supernatant was then subjected to tangential flow filtration (Pall) to buffer exchange into 20 mM phosphate, pH 7.4, 250 mM NaCl, 20 mM imidazole, and 0.005% Tween-20. To purify the PfRh2b protein, Ni^2+^-NTA (HisTrap FF; GE Healthcare) chromatography was followed by gel filtration on a Superdex 200 16/60 column. The protein was concentrated using an Amicon centrifugal filter device (MW cut-off, 10 kDa).

### Enzyme-linked immunosorbent assay

To measure IgG response against PfRh2b and PfRh5, 96-well ELISA plates (Maxisorp, NUNC, Denmark) were coated with the recombinant proteins at 1 µg/mL overnight at 4 °C and blocked with 5% non-fat milk in phosphate buffered saline (PBS) for 1 h. Plates were then incubated with 100 µL/well of test plasma samples diluted 1:500 in 1% non-fat milk in PBS. A pool of semi-immune sera diluted 1:50 and titrated 3-fold over seven duplicate wells on each plate was used as a standard calibrator. Plasma samples were incubated for two hours at room temperature and plates were afterwards incubated with 100 µL/well of horseradish peroxidase-conjugated goat anti-human IgG (KPL), diluted 1:1000 in 1% non-fat milk, for 1 hour. In between the different incubation steps, plates were washed five times with PBS, pH 7.4, 0.05% Tween 20 using an automated plate washer. This was followed by a colour development step with 100 µL/well of TMB substrate (KEM-EN-TEC, Taastrup, Denmark) for 10 minutes and the colour reaction was stopped by adding 50 µL/well of 0.2 N H_2_SO_4_. Optical densities (ODs) were subsequently read at 450 nm using a 96-well ELISA plate reader (BioTek, VT, USA). ODs from each plate were converted into antibody units with the calibrator data using the four-parameter logistic fit program known as the Auditable Data management System for ELISA (ADAMSEL version 40, Edmond J. Remarque^®^).

## Supplementary information

Supplementary information.
