# Supplementary material for: Analysis of Plasmodium falciparum Rh2b deletion polymorphism across different transmission areas
Source: Sci Rep. 2020 Jan 30;10:1498. doi: 10.1038/s41598-020-58300-3 (PMC6992740; doi:10.1038/s41598-020-58300-3)
Supplement: Supplementary file 1 — Supplementary information. [file 41598_2020_58300_MOESM1_ESM.pdf]

Analysis of *Plasmodium falciparum* Rh2b deletion polymorphism across different transmission areas

Yaw Aniweh[1, 2*], Jonathan Suubaar[1,2#], Collins M. Morang'a[1,2#], Prince B. Nyarko[1,2], Katherine E. Wright[3,4], Kwadwo A. Kusi[1,2,5], Felix Ansah[1,2], Eric Kyei-Baafour[5], Evelyn Quansah[1,2], Jessica Asante[5], Laty Gaye Thiam[1,2], Matthew K. Higgins[3], Gordon A. Awandare[1,2*]

[1]West Africa Centre for Cell Biology of Infectious Pathogens, [2]Department of Biochemistry, Cell and Molecular Biology, College of Basic and Applied Sciences, University of Ghana, Accra, Ghana

[3]Department of Biochemistry, University of Oxford, South Parks Road, Oxford, OX1 3QU, UK

[4]Department of Life Sciences, Imperial College London, London, UK

[5]Immunology Department, Noguchi Memorial Institute for Medical Research, College of Health Sciences, University of Ghana, Legon, Ghana

*For Correspondence contact yaniweh@ug.edu.gh or gawandare@hotmail.com

[#]Authors contributed equally

Supplementary information legend

Supplementary Figure 1: Sequence alignment showing PfRh2b deleted. An alignment of sequences showing the region for the PfRh2b deletion among P. falciparum clinical isolates from Ghana. Chromosome loci is indicated in numbers above.

Supplementary Figure 2: Representative gel showing clonality of the mixed parasites. The different parasites used for the assay labelled at the top of the gel. Molecular weight marker indications on the right

Supplementary table 1: PfRh2b deletion frequencies in different communities in Ghana

Supplementary Figure 1

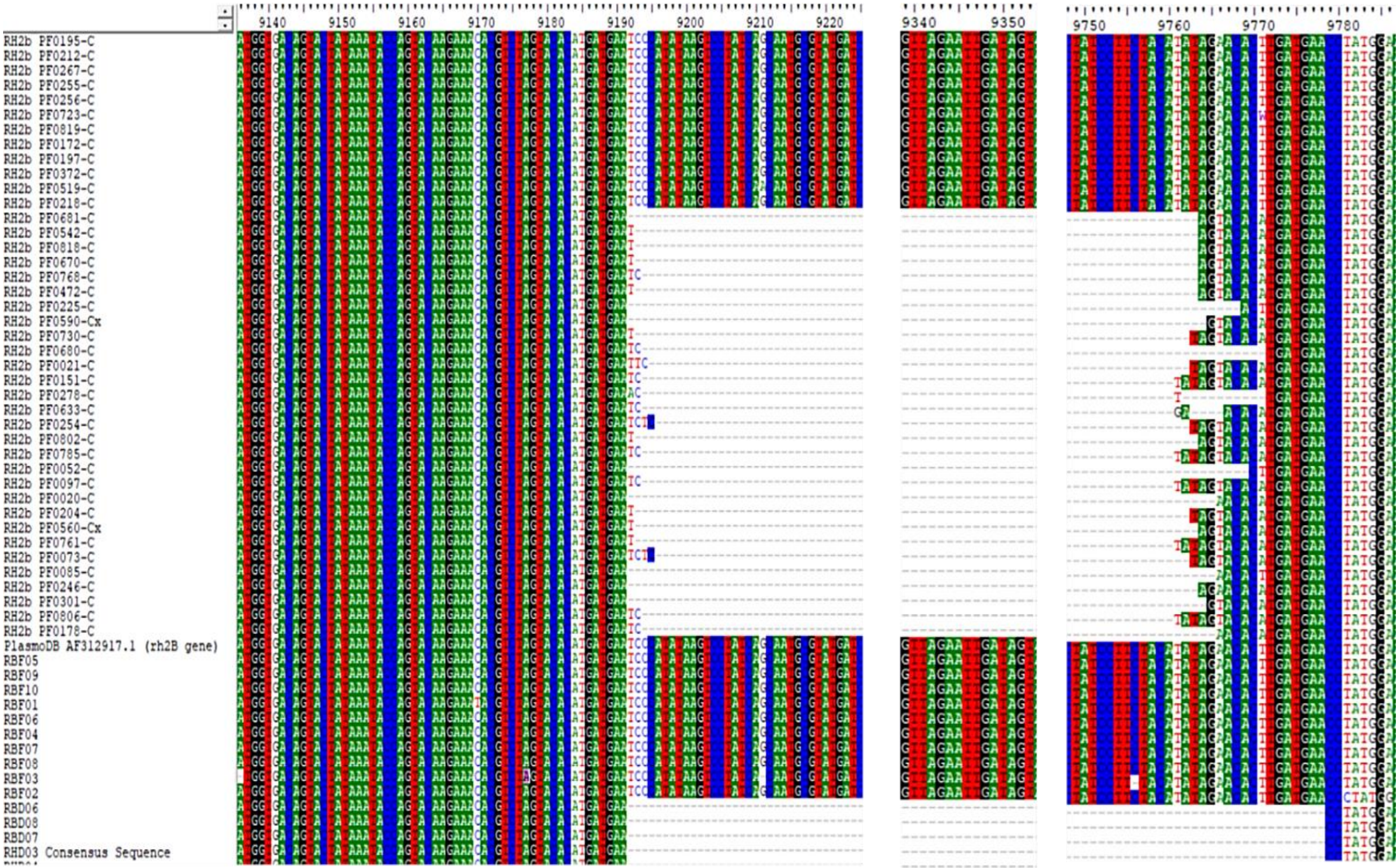

Supplementary Figure 2

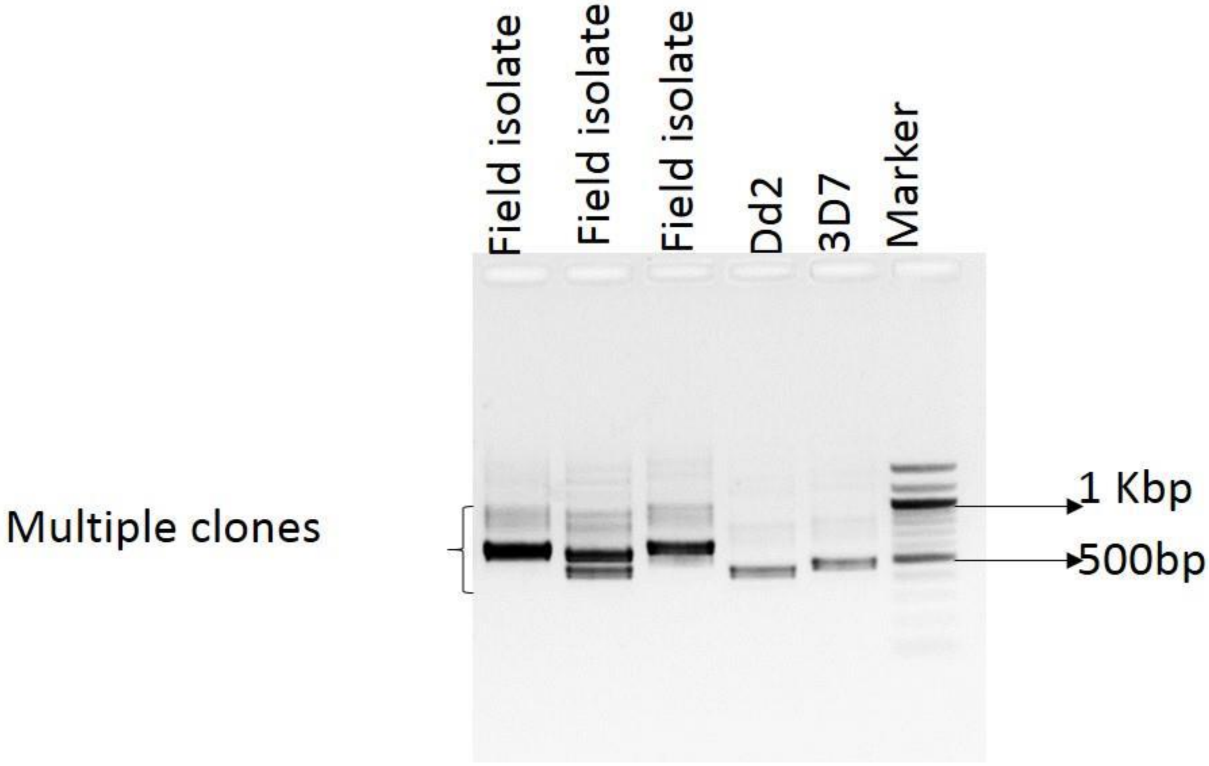

Supplementary Table 1: Deletion frequency of PfRh2b in three sample collection sites in Ghana

Gene	Allele	Accra	Cape Coast	Kintampo	Sogakope	X^2 (df)	*p-value*
**PfRh2b**	Full Length, N (%)	74 (47.74)	264 (42.72)	104 (41.94)	106 (53.81)	5.95 (3)	0.114
	Deletion, N (%)	78 (50.32)	296 (47.90)	132 (53.23)	85 (43.15)		
	Mixed, N (%)	3 (1.94)	58 (9.38)	12 (4.83)	6 (3.04)		
